# Motivations and experiences of patients with respiratory disease and their caregivers in a multinational trial of mirtazapine for severe breathlessness: a qualitative study (BETTER-B)

**DOI:** 10.1186/s12890-025-03862-z

**Published:** 2025-08-14

**Authors:** Adejoke O. Oluyase, Luca Ghirotto, Harry Watson, Massimo Costantini, Sabrina Bajwah, Charles Normand, Silvia Tanzi, Jeremias Bazata, Karen Ryan, Elena Turola, Irene J. Higginson, Matthew Maddocks, On behalf of the BETTER-B consortium, Miriam J. Johnson, Simon P. Hart, Charlotte E. Bolton, Julia M. Brown, Hannah Mather, Deb Smith, Claudia Bausewein, Kathrin Kahnert, Steffen T. Simon, Małgorzata Krajnik, Piotr Janowiak, Ewa Jassem, Emer Kelly, Samantha Smith, Mirco Lusuardi, Rossella Cianci

**Affiliations:** 1 Florence Nightingale Faculty of Nursing, Midwifery & Palliative Care, Cicely Saunders Institute of Palliative Care, Policy and Rehabilitation, King’s College London, Bessemer Road, London, SE5 9PJ UK; 2 Azienda USL-IRCCS di Reggio Emilia, Reggio Emilia, Italy; 3 King’s College Hospital NHS Foundation Trust, Bessemer Road, London, UK; 4 Department of Palliative Medicine, LMU University Hospital, LMU Munich, Munich, Germany; 5 University College Dublin and Mater Misericordiae University Hospital Dublin, Dublin, Ireland

**Keywords:** Antidepressant, Breathlessness, Caregivers, Experiences, Mirtazapine, Motivations, Patients, Qualitative, Repurposing

## Abstract

**Background:**

Drug repurposing offers advantages over traditional drug development, such as shorter time and reduced costs. Understanding patient and caregiver perspectives on repurposed medicines is crucial to improving clinical trial design and conduct, especially in advanced disease. We carried out this study in the UK and Italy to explore the experiences and motivations of patients with respiratory diseases and their caregivers who participated in a trial investigating the repurposing of the antidepressant mirtazapine to alleviate severe breathlessness.

**Methods:**

Qualitative study nested within a double-blind, placebo-controlled, randomised trial (BETTER-B: BETter TreatmEnts for Refractory Breathlessness). Purposive sampling ensured diversity in age and gender. Framework analysis was applied. Interviewed participants had Chronic Obstructive Pulmonary Disease (COPD) or Interstitial Lung Disease (ILD), experienced limiting breathlessness (grade 3 or 4 of the modified Medical Research Council breathlessness scale) and had participated in the trial.

**Results:**

Twenty-three patients (13 men and 10 women) and eight caregivers (4 men and 4 women) were interviewed. Of patients (15 COPD, 8 ILD), 22 had completed the trial and one had withdrawn due to adverse effects. Interviews were conducted at home or via the telephone. Two main themes were derived: (1) 'knowledge and views about antidepressants and its use’ and (2) ‘experience and views on joining the trial’. The patients’ perceived need for relief from severe breathlessness and its impact outweighed any concerns about taking an antidepressant. Motivations for trial participation included the potential for benefit, altruism, trust in the healthcare system, and the strength of relationships between patients and healthcare professionals.

**Conclusions:**

Participants willing to accept mirtazapine repurposed for another indication joined this trial regardless of their existing concerns about antidepressants. A clear explanation of trials and possible benefits, plus trust in professionals and the healthcare system, are instrumental in increasing trial participation for repurposed medicines.

**Trial registration:**

ISRCTN Registry, ISRCTN10487976. Prospectively Registered on 19 November 2019.

**Supplementary Information:**

The online version contains supplementary material available at 10.1186/s12890-025-03862-z.

## Background

Breathlessness is a prevalent and burdensome symptom in chronic respiratory diseases, and few pharmacological treatment options are available [1]. Severe breathlessness is also associated with increased healthcare utilisation [2, 3]. Low-dose, sustained-release morphine is the only approved treatment for severe breathlessness, and even this is limited to Australia [4]. Furthermore, the use of morphine to treat breathlessness is only supported by weak evidence showing a small effect [5]. Given the need for new therapies, drug repurposing—using existing medicines for new indications—offers a promising approach, reducing development time and costs [6, 7].

Clinical trials are needed to determine the effectiveness of medicines for their alternative proposed indications. Mirtazapine, a widely used antidepressant, is thought to modulate respiratory sensation and thereby might alleviate severe breathlessness by enhancing neurotransmitter levels like serotonin [8, 9]. Although mirtazapine is sometimes prescribed for breathlessness, based on case and feasibility reports, its effectiveness, cost-effectiveness, and safety remain unproven, necessitating further investigation through a randomised, double-blind clinical trial [10].

People participate in clinical trials for different reasons, including personal benefits, trust in their clinicians, closer monitoring, better quality care, and helping society and others [11–14]. Trials of repurposed medicines present challenges. Although their safety profile is well-established from their licensed use, efficacy for their new indication is not established [7]. This uncertainty about the efficacy of repurposed medicines potentially limits early access to effective treatment through trial participation.

Furthermore, the impetus to carry out trials by clinicians and patients’ willingness to participate in trials may be low in advanced diseases, with clinicians opting to try treatments without thorough evaluation due to a desire to relieve suffering in patients with poor prognoses and minimal treatment options [15–17]. This is evident by the high levels of off-label medicines use for severe breathlessness and in palliative care more widely [15, 17].

Concerns may exist due to the public's understanding of the medicine. Stigma surrounding some medicines, such as antidepressants, may also influence people’s decision-making regarding trial participation [18]. Caregivers play a pivotal role in managing breathlessness, supporting patients, and providing information on the benefits and adverse effects of treatment [19]. Little is known about the experiences and motivations of patients and caregivers in clinical trials of repurposed medicines. Therefore, we embedded qualitative interviews with patients with Chronic Obstructive Pulmonary Disease (COPD) and Interstitial Lung Disease (ILD) and their caregivers within a randomised trial.

We aimed to explore the experiences and motivations for participating in a clinical trial of mirtazapine, an antidepressant, repurposed for severe breathlessness in people with respiratory diseases. Our objectives were.

i) to understand the acceptability, tolerability and compliance of the trial medication and.

ii) to identify the potential facilitators and barriers to the medicine uptake.

## Methods

### Study design

We conducted a qualitative interview study embedded within the BETTER-B trial. BETTER-B was an international, multicentre, phase 3, parallel-group, double-blind, randomised, placebo-controlled pragmatic trial of mirtazapine for severe breathlessness [10]. It was carried out in 16 centres across seven countries. Trial registration: ISRCTN10487976, ISRCTN15751764 (Australia/New Zealand) EudraCT 2019–002001-21. The qualitative study’s protocol, the written informed consent form, and other materials related to the participants were approved by the UK (REC reference: 21/LO/0844) and Italian ethics committees (in-house prot. n. 248/2022/OSS/IRCCSRE). The trial was co-sponsored by King’s College London and King’s College Hospital. This study is reported in accordance with the consolidated criteria for reporting qualitative studies (COREQ) guidance [20].

### Setting and participants

Consenting trial participants were approached for a qualitative interview study at the end of trial participation or withdrawal in one UK and one Italian centre. Due to funding and resource constraints, these two centres were purposively sampled among the participating centres.

Patients who consented to trial participation were asked to identify the person closest to them, who was also approached to consent to data collection in the trial. Patients did not have to identify a caregiver to participate in the trial. Eligible participants in the qualitative study had taken part in the BETTER-B trial [10], could comply with interview requirements and provide written informed consent. Participants (who had consented to the trial) took part in the interviews. They were purposively sampled by age, education, gender, income, marital status and living arrangement to ensure diversity. From the study design to analysis, patient and public involvement (PPI) members were involved.

### Data collection and processing

Interviews were scheduled where convenient after written informed consent had been obtained between March 2022 and October 2023. We conducted face-to-face or telephone semi-structured interviews with participants and their caregivers and collected data on their narratives and experiences with trial participation and the medication. At the time of the interviews, participants did not know which treatment arm they were allocated to. Section I of the Supplementary Material shows the topic guides used for the patient and caregiver interviews. The topic guide was translated into Italian for the Italian participants. Interviews were conducted in English (UK) by AO (a female researcher) and HW (a male researcher), and in Italian (Italy) by LG (a male researcher). AO and LG are experienced qualitative researchers with PhDs. HW, a novice qualitative researcher with a background in Chemistry, was trained and supervised by AO. All but one interview was audio recorded. Interviews were transcribed verbatim in the native language by the researchers and a transcription company. Field notes were completed to describe the flow of the interview, contextual factors and personal reflections.

### Data analysis

Interviews were analysed using framework analysis [21]. As framework analysis can be considered an interpretative process [21], the analyses may have been influenced by the researchers’ professional experiences. However, in qualitative research, the researcher’s subjectivity is often regarded as a valuable resource. Full transcripts were coded by AO, LG and HW. Data analysis was supported using Microsoft Excel. AO and HW coded the English transcripts while LG coded the Italian transcripts. We adapted and revised a pre-existing framework [22] from relevant literature as a starting point for our analysis. Using the framework, we conducted an initial round of analysis, focusing on predetermined categories and themes. The analysis involved the following stages: transcription, familiarisation, coding, developing the analytical framework, applying the analytical framework, charting data into the framework matrix and interpreting the data [23]. We refined the framework to capture new insights as the analysis progressed. AO, HW, LG and MM led the analysis, but the interpretation was collaborative, iterative and reflexive, drawing on the research team’s (MC and ET) diverse perspectives and experiences over several meetings. AO, HW, MM and MC worked as researchers in palliative care when analyses were performed. The themes and coded extracts were shared with the breathlessness PPI group for feedback and insight.

## Results

Thirty-one participants were interviewed: 17 patients and 5 caregivers in the UK, and 6 patients and 3 caregivers in Italy, with three Italian patients interviewed with their caregivers. Of the 31 participants, 17 were men and 14 were women. Nine participants identified as white Italians, 20 as white British, one as other white background, and one as Caribbean.

Most participants were aged between 65 and 84 (Table 1). The mean interview duration was 27 min (13–47 min). Eleven patients received mirtazapine, and 12 patients received a placebo.

**Table 1:** Characteristics of interviewed participants (patients and caregivers)

Characteristics		Italian Patients *n* = 6	Italian Caregivers *n* = 3	UK Patients *n* = 17	UK Caregivers *n* = 5
Gender	Male	4	1	9	3
	Female	2	2	8	2
Age	55–64	0	1	2	1
	65–74	4	2	4	1
	75–84	2	0	8	3
	> 85	0	0	3	0
Ethnicity	White (Italian)	6	3	0	0
	White British	0	0	16	4
	Other White Background	0	0	0	1
	Caribbean	0	0	1	0
Marital Status	Married/civil partnership/relationship akin to marriage	6	3	10	4
	Divorced/separated	0	0	3	0
	Widowed	0	0	4	0
	Single	0	0	0	1
Living with	Alone	0	0	5	0
	Spouse/partner	6	3	9	3
	Children	0	0	1	0
	Spouse/partner/children	0	0	1	1
	Others	0	0	1	1
Education	Less than < = 15 years	3	2	8	3
	16/17 years	0	0	4	1
	18/19 years	2	1	0	1
	Diploma/professional school diploma	1	0	0	0
	University	0	0	5	0
Income	I live comfortably on my current income	1	1	8	1
	I meet expenses with my current income/coping on present income	4	1	6	3
	Difficult on present income	0	0	2	1
	Very difficult on present income	0	0	1	0
	Prefer not to answer	1	1	0	0
Relationship with patient	Spouse/Partner	NA	3	NA	4
	Son/daughter	NA	NA	NA	1
Employment status	Retired	NA	3	NA	5

*NA *Not Applicable

Participants’ experiences of the trial medicines and motivations for trial participation are presented in Table 2. We developed two main themes: knowledge and views about antidepressants and their use, as well as experience and views on joining the trial.

**Table 2:** Main themes and subthemes from the interviews

Main themes	Sub-themes
Knowledge and views about antidepressants and its use	Prior experience with antidepressants
	Knowledge of mirtazapine
	Views about using antidepressants for breathlessness
	Adherence to trial medicine
Experience and views on joining the trial	Patient/caregiver expectations
	Motivations for trial participation and use of trial medicine
	Information/communication
	Knowledge of placebo

## Main theme 1: Knowledge and views about antidepressants and its use

This theme was developed in response to addressing our first objective. Participants displayed a willingness to accept an antidepressant for breathlessness if it could improve their quality of life or help with their specific condition. Participants'knowledge and views regarding antidepressants and its use varied widely.

This theme comprised four sub-themes.

### Prior experience with antidepressants

Most participants reported limited or no personal experience with antidepressants. However, a minority described use of antidepressants for depression or other indications and the reservations they had when commencing the trial:

…I think, when I entered the trial, one of the concerns I had expressed was that it was an antidepressant. I had had bad experience once with an antidepressant that was for other reasons, that my GP had prescribed for me, so that was the major reservation I had about embarking on or joining the trial, was the fact that it was an antidepressant (Patient 199, ILD, male, UK)

While some participants lacked detailed knowledge about antidepressants, they were open to discussing these medications and their potential side effects, indicating a degree of curiosity and a willingness to learn and use them during the trial.

### Knowledge of mirtazapine

Participants’ understanding of mirtazapine’s purpose and intended effects varied. Some had limited knowledge while others had a better grasp of the medication.

Nothing, personally. I don't know anything about it, to be honest. I don't know what it's meant to be doing for [patient]. (Caregiver 161, female, UK)

So, they told us that this medication is currently prescribed to people with burnout, depression; so it is available for these conditions. However, in our case, they would recommend it because having this state of dyspnea, it could probably also act on dyspnea. (Patient PZ05, ILD, male + Caregiver PZ05 female, Italy)

Some participants conducted internet research to learn more about mirtazapine after enrolling in the trial, suggesting that their knowledge about the medication was primarily acquired during the trial.

### Views about using antidepressants for breathlessness

Participants generally demonstrated a pragmatic and open-minded approach to accepting antidepressants for the trial, prioritising potential benefits over the stigma associated with mental health issues or personal biases.

I just need something to help me breathe, that's all. (Patient PZ01, COPD, female, Italy).

I didn’t want to take an antidepressant for depression (be)cause I’m not depressed. I think, if I was asked if I wanted to take an antidepressant I would probably say no simply because I'm inundated with pills anyway and I’m not depressed, but if someone tell me to take because it has another effect, then I might, if I was told that in the long term situation it was going to help my breathing I would definitely take them. (Patient 71, COPD, female, UK)

Some participants raised concerns about the potential for addiction to antidepressants but believed their need for relief outweighed this concern.

Well, I'm always a bit nervous of addictive medicines. It's not something I'd particularly steer towards myself if I didn't need to. (Patient 104, ILD, male, UK)

### Adherence to trial medicine

Participants generally adhered to the study medicine regimen, motivated by their commitment to the trial and the hope of experiencing relief from their symptoms. They diligently followed the dosage instructions and maintained consistency in taking the medication.

I took it religiously every night. Even if sometimes it was a bit late, I still took it religiously. (Patient 65, COPD, female, UK)

A few participants described forgetting to take the medication or missing the dose tapering. Some expressed concerns about using the trial medicines with other medicines they were taking. One participant reported side effects and her decision to stop taking the medicine.

I think I only took one or two because it made me feel awful. Terrible, actually. (Patient 124, COPD, female, UK)

## Main theme 2: experience and views on joining the trial

The initiation phase of the clinical trial was crucial, as it captured the participants'decision-making processes regarding enrolment, including possible facilitators and barriers to trial engagement and uptake of mirtazapine. This theme provides insights into participants and their caregivers’ expectations, motivations, the communication they received, and their understanding of the placebo concept.

### Patient/caregiver expectations

Participants expressed a range of expectations for taking part in the trial and use of the trial medicine, primarily centred around improving their breathing, functioning and overall quality of life. For instance, one caregiver hoped that participation might ease their spouse's physical mobility:

Well, I would have thought making breathing easy, easier. And maybe going to some length of clearing up the infection, or whatever it is… Well, I suppose in the sense of perhaps making it easier for my wife to get around physically. (Caregiver 124, male, UK)

Participants generally exhibited a sense of realism, acknowledging limited expectations while others did not expect a “miracle cure”.

No, I didn't expect anything, you see. I did it because if it helps, I'm happy to do it, but I didn't expect to find a miracle cure. I'm very realistic, you know (Patient PZ 01, COPD, female, Italy)

### Motivations for trial participation and use of trial medicine

Participants expressed varied reasons for trial participation, including potential benefits, following their clinician’s recommendations and contributing to science and the society. For some, desperation for any potential relief was a driving force:

When there's a need, you try everything. Afterward, they had me take this pill. (Patient PZ04 COPD, male, Italy)

Others were motivated by a sense of duty and past positive experiences with medical research.

The altruistic desire to help others and the healthcare system were also significant motivators, based upon trust in clinicians and the healthcare system:

Basically, a lot of people expect a lot from the NHS. If there is anything I can do personally to help other people or indirectly myself in the future I’d like to do it. We can’t complain about different things within the NHS if we are not prepared to step up and do something. In effect they need help because they need people to test the drugs on, don’t they? (Patient 119, COPD, male, UK)

Some participants questioned their participation as they did not notice any benefit from the trial medicine:

Sometimes I had reached the point where I thought,"Why am I going there? What am I doing?"Going all the way to [research hospital] for what? I mean, I know it's free, but how much longer? I have travel expenses, so it's an extra cost that I don't see the utility of. I thought about it, but then I started, and I followed through (Patient PZ 03, COPD, male, Italy)

A participant described how she negotiated taking reduced alcohol quantity on the trial without which she would not have considered participating:

…every week we had a guest speaker – the guest speaker talked about this trial, and asked if anyone was willing. I said I was, but then I said I drank two gin and tonics a day, six days a week, and we had to do a bit of to-ing and fro-ing to accommodate that (Patient 173, COPD, female, UK).

### Information/communication

Participants reported receiving information about the trial through verbal and written communication. Some found the information provided adequate, while others felt the information could have been more detailed, particularly regarding potential side effects:

Well, I think it was verbal and written…Perhaps a touch more details about the possible side effect. Well, I mean, we were told there were side effects, but I’m not sure we were told exactly what that would mean. (Caregiver 124, male, UK)

### Knowledge of placebo

Many participants had a basic understanding of placebos, describing them as "false drugs" or "dummy tablets". They generally understood that placebos did not contain the active ingredient of the real drug.

Uncertainty about whether they had received the actual drug or a placebo was common:

I've always thought that a placebo was a replica of the real drug, but I've got no idea whether I was on the real drug or the placebo myself. (Patient 104, ILD, male, UK)

## Discussion

### Summary of study findings

This study explored the experiences and motivations of participants with respiratory diseases participating in a clinical trial of a repurposed antidepressant for severe breathlessness. Participants’ determination to seek relief from breathlessness and its impact on their lives generally outweighed any negative views about antidepressants. We found that most participants were willing to accept a known antidepressant for breathlessness.

### Findings in relation to existing literature

Participants’ acceptance of antidepressants was hinged on desperation for relief from their symptoms and is consistent with findings from a previous study of factors influencing drug trial participation in people with chronic or refractory breathlessness and advanced disease [24]. A recent meta-synthesis looking at antidepressant use for depression also showed similar findings [25]. Furthermore, a study that explored patients and their caregivers’ experiences of morphine in an RCT for breathlessness management reported improvement in function as a key consideration in taking opioids, with symptom reduction and adverse effects influencing opioid use during and after the trial [26].

While some participants reported lacking detailed knowledge about antidepressants and placebo, they were open to discussing these medications and participating in the study. Although the desire to use off-label medicines by clinicians may be high when treating difficult symptoms like breathlessness within the context of poor prognosis and very limited treatment options [15–17], it is important to offer patients and their families clear information and opportunities to be involved in trials. Involvement of those with advanced illnesses in research often raises ethical challenges relating to vulnerability, capacity issues and potential for benefit or harm [27]. Consequently, careful consideration must be given to providing extra support or tailoring clinical trial processes to the needs of participants in order to safeguard autonomy and assure beneficence, non-maleficence, and justice [27].

The influence of potential participants’ knowledge of the study and its processes on trial participation has been found to be mixed [28, 29]. Knowledge could be a barrier when information is considered to be vague [30] or complex [31, 32]. The reasons for participation in this clinical trial are consistent with the literature [11, 24, 28, 33]. These included the potential for personal benefit, the desire to contribute to research, trust in their clinicians and the healthcare system and altruism. Lack of trust in the healthcare system can be a deterrent to research participation as patients may be suspicious of the motivation for research [14].

Although drug repurposing has its benefits [6, 7], concerns may arise because of potential adverse effects, uncertain effectiveness when used off-label and its impact on clinical trial participation. Previous studies have shown that the fear of addiction, dependence or other adverse effects may affect whether patients take antidepressants or seek help [34, 35]. While 100 (18%) of 557 eligible participants did not want to receive mirtazapine and as a result, declined trial participation, most eligible patients approached were willing to participate in this study in spite of any concerns [10]. Of the 1919 ineligible participants excluded from the trial, 482 (25%) were already on antidepressants (e.g. mirtazapine) or other serotonergic active substances. It appeared some clinicians were already prescribing mirtazapine off-label without thorough evaluation in a clinical trial. Clinicians should advocate for increased research into the effectiveness and safety of new medicines aimed at alleviating breathlessness in palliative care and respiratory medicine.

In contrast to a previous overview of systematic reviews of psychosocial barriers and facilitators to research participation –which identified placebo use as a barrier [28], participants in this trial accepted the use of a placebo, with some even perceiving it to have an effect. Although placebos are considered inert substances, they can influence medical and psychological outcomes. As participants were blinded in this trial, it is unsurprising that they generally reported being unaware of their treatment allocation. Furthermore, the BETTER-B trial found no evidence of a clinical improvement in severe breathlessness with mirtazapine compared to placebo [10]. This lack of benefit might also contribute to the success of blinding of trial participants. The involvement of caregivers in the study was important as they supported participants with severe breathlessness. Caregivers often help patients with daily activities and emotional functioning [19, 36].

### Practical recommendations for future trial recruitment strategies

The learning from this study could be used to try to address concerns among those in the target population, e.g. through clear and reassuring trial advertisements. The manner in which a potential study participant is approached—particularly in the context of drug availability, public concern, and stigma, as seen with antidepressants—can influence their decision to participate. A trusting relationship with healthcare professionals can facilitate research participation and patient-centred care [28, 37], including in drug trials of chronic or refractory breathlessness [24]. A listening ear, being approachable, and interpersonal skills have been identified as being essential for palliative care professionals [24]. There is a need for training of research staff in this area and effective communication [38] to increase uptake of clinical trials by patients with advanced diseases and their caregivers..

The above findings should be considered when planning and setting up palliative care drug trials to ensure optimal enrolment of participants. Although concerns have been raised about including patients with advanced diseases in drug trials, our findings and the existing evidence [24, 39] suggest that this patient population want the opportunity to be involved in research.

### Strengths and limitations

This qualitative study explored why participants across two countries decided to participate in a clinical trial of mirtazapine repurposed for breathlessness. We carried out in-depth interviews with UK and Italian participants. Fourteen of the interviewees were men, which is representative of the trial population and similar to other trials [24, 33, 40], likely reflecting that chronic lung disease had mostly affected men [41]. The difference in the prevalence of lung diseases between men and women has diminished in many countries [42]. We did not achieve diversity in socioeconomic factors such as ethnicity and income. Although the lack of diversity in ethnicity reflects the demographic composition of the population who took part in the trial, it implies that our findings may not fully capture the views of those from minoritised communities. People from minoritised communities such as Asian and Black ethnic groups are less likely to participate in research than their white counterparts due to cultural beliefs related to health, language and mistrust of medical research and professionals [43, 44]. It is recognised that researchers have failed to reach these underserved populations due to culturally inappropriate recruitment procedures and lack of adequate engagement [45]. The representation of those from minoritised communities in chronic lung disease trials should be a priority for future studies.

Three researchers (two UK and one Italian) conducted all the interviews, with double coding of a subset of transcripts and regular discussions with the research team to minimise interpretation bias. Social desirability bias is possible as participants may have been reluctant to criticise the trial intervention and processes. Recall bias was likely due to the time interval between ending the trial and participating in the qualitative interview. Furthermore, there is the possibility of selection bias as we only interviewed those who had taken part in the BETTER-B trial, and only one interviewed participant withdrew due to adverse effects.

## Conclusion

Participants' willingness to accept an antidepressant for severe breathlessness and take part in a clinical trial was influenced by their urgent need to seek relief from this challenging symptom and improve their functioning. Altruism also played a role. A trusting relationship with healthcare professionals, clear communication of the benefits of participation, and strategies to increase trust in the healthcare system may help increase involvement in trials of repurposed medicines in palliative care.

## Supplementary Information

Supplementary Material 1.

## Data Availability

The datasets generated and analysed during the current study are not publicly available due to participants’ privacy and the sensitive nature of collected data, but they are available from the BETTER-B consortium led by Professor Irene J. Higginson upon reasonable request.
