# Supplementary material for: Motivations and experiences of patients with respiratory disease and their caregivers in a multinational trial of mirtazapine for severe breathlessness: a qualitative study (BETTER-B)
Source: BMC Pulm Med. 2025 Aug 14;25:390. doi: 10.1186/s12890-025-03862-z (PMC12351812; doi:10.1186/s12890-025-03862-z)
Supplement: Supplementary file 1 — Supplementary Material 1. [file 12890_2025_3862_MOESM1_ESM.docx]

**Supplementary Appendix**

**Motivations and experiences of patients with respiratory disease and their caregivers in a multinational trial of mirtazapine for severe breathlessness: a qualitative study (BETTER-B)**

Oluyase, Adejoke O; Ghirotto, Luca; Watson, Harry; Costantini, Massimo; Bajwah, Sabrina; Normand, Charles; Tanzi, Silvia; Bazata, Jeremias; Ryan, Karen; Turola, Elena; Higginson, Irene J; Maddocks, Matthew; BETTER-B consortium.

**
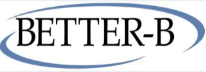
This project has received funding from the European Union’s Horizon 2020 research and innovation programme under grant agreement No 825319**

**
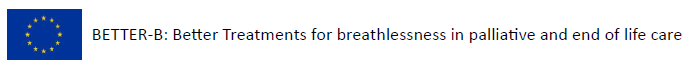
**

Project Website: <https://betterbreathe.eu>

**Contents**

[Section I: Topic guide 2](#_Toc181115725)

[Section II. BETTER-B Research Consortium and Acknowledgements 6](#_Toc181115726)

[Consortium members 6](#_Toc181115727)

[Acknowledgements 8](#_Toc181115728)

# **Section I: Topic guide**

**Interview topic guide (patients)**

You have recently taken part in the BETTER-B trial. I would like to talk to you to understand your experience of taking part, what you expected, and what it was like.

Are you happy for the interview to be audio recorded today? And for me to make notes during the interview? The recording will be typed up, but all names or places that could identify you, or other people will be removed. Once we have typed the interview, we destroy the recording.

We may publish quotes from your interview, but we use an identification number to ensure you cannot be identified. Is this okay?

Do you have any questions before we begin?

If you want to stop the interview at any point, please let me know.

**Introduction/ BETTER-B**

- What do you know about the drug being studied?

(prompt: mirtazapine is an antidepressant)

- What other medications do you take?

(prompt: what are these for?)

**Attitudes and beliefs about antidepressants**

- How do you feel about taking an antidepressant for breathlessness?

(prompt: did you have any concerns about the drug, personal views about those who take antidepressants, side effects, addiction, long-term effects, what others would think of you, religious views)

- Did any these [as discussed above] influence whether you continued or thought about stopping taking the trial drug?
- What do you understand about a placebo drug?

**Facilitators and barriers to uptake of antidepressants**

- Could you please me tell me how you managed taking the trial drug?

(prompt: any difficulties, tablet size, frequency)

- Do you have any situations or factors that make it hard for you to take your trial drug?
- How did you manage with your other medications?

(prompt: use of a Dosette Box, Diary reminder)

- What situations or factors make it easy for you to take your trial drug?

(prompt: can you tell me more about that?)

**Information, communication and influence in decision to take part**

- At the start of the study, you were given information about the trial drug. Was this information adequate?
- If not, what more would you like to know?
- How would you like to receive this information?
- How were you asked to take part in the study? What was that like?

(prompt: Who spoke to you? What were you told? Where were you at the time? What were your expectations?)

- Why did you decide to take part?

(prompt: wanting to help others, see improvement in symptoms)

- (If withdrawer: could you please tell me about your decision of withdrawing the trial?)

**Perceived benefit vs burden**

- Before starting the study, what did you hope would change by taking part? What was the most important change you were hoping for?
- Did the trial drug change the way you felt? If yes: in what ways?

(prompt: did you notice any change in your breathing, sleep, appetite, drowsiness, mood?)

- What do these changes mean for you day to day, week to week (social, function)?
- Were there any changes you had not expected?

**Experience of side effects (including stopping, titration)**

- Did you stop taking the trial drug or reduce the dose because of side effects? If yes: what were the side effects and how did they impact on your life?

**Closing section**

- Is there anything else you would like to tell me?
- Is there anything that has worried you during this conversation?

**Interview topic guide (caregivers)**

You have recently taken part in the BETTER-B trial as a caregiver or family member. I would like to understand your experience of taking part and your perspective about what it was like for [NAME of family member/friend].

Are you happy for the interview to be audio recorded today? And for me to make notes during the interview? The recording will be typed up, but all names or places that could identify you, or other people will be removed. Once we have typed the interview, we destroy the recording.

We may publish quotes from your interview, but we use an identification number to ensure you cannot be identified. Is this okay?

Do you have any questions before we begin?

If you want to stop the interview at any point please let me know.

**Introduction/ BETTER-B**

- What do you know about the drug being studied?

**Attitudes and beliefs about antidepressants**

- How do you feel about [NAME of family member/friend] taking an antidepressant for their breathlessness?

(prompt: did you have any concerns about the drug itself (e.g. personal views about those who take antidepressants, side effects, addiction, long-term effects, what others would think, religious views)

- What do you understand about a placebo drug?

**Facilitators and barriers to uptake of antidepressants**

- Did [NAME of family member/friend] experience any difficulties or problems with the trial drug?

(Prompt: tablet size, frequency)

- Have they asked for your assistance? If yes: could you tell me what do you do? What factors make it easier for you to assist them?

**Information and communication**

- At the start of the study you were both given information about the trial drug. Was the information adequate?
- If not, what more would you like to know?
- How would you like to receive this information?

**Perceived benefit vs burden**

- Before starting the study, what did you hope would change by taking part? What was the most important change you were hoping for?
- Did the trial drug change the way [NAME of family member/friend] felt? If yes: tell me in what ways

(prompt: Did you notice any change in his/her breathing, sleep, appetite, drowsiness)

- What did these changes mean for you day to day, week to week (social, function)?
- Were there any changes you had not expected?

**Experience of side effects (including stopping, titration)**

- Did [NAME of family member/friend] stop taking the trial drug or reduce the dose because of side effects? If yes: what were the side effects and how did they impact on your lives?

**Closing section**

- Is there anything else you would like to tell me?
- Is there anything that has worried you during this conversation?

# Section II. BETTER-B Research Consortium and Acknowledgements

| Consortium members |  |  |  |  |  |
| --- | --- | --- | --- | --- | --- |
| Key role | Title | First name | Surname | One degree | Organisation, country |
| Chief Investigator | Professor | Irene J | Higginson | FFPHM | KCL, UK |
| Project Manager | Dr | Adejoke O | Oluyase | PhD | KCL, UK |
| Co-Investigators/grant holders | Professor | Matthew | Maddocks | PhD | KCL, UK |
| Co-Investigators/grant holders | Dr | Massimo | Costantini | MD | KCL, UK |
| Co-Investigators/grant holders | Dr | Sabrina | Bajwah | PhD | KCL, UK |
| Co-Investigators/grant holders | Professor | Charles | Normand | DPhil | KCL, UK |
| Research Assistant | Mr | Harry | Watson | BSc | KCL, UK |
| Co-Investigators/grant holders | Professor | Claudia | Bausewein | PhD | UMUEN, Germany |
| Co-Investigators/grant holders | Professor | Steffen T | Simon | PhD | KOELN, Germany |
| Co-Investigators/grant holders | Professor | Karen | Ryan | MD | UCD, Ireland |
| Site Principal Investigator | Professor | Miriam J | Johnson | MD | Hull, UK |
| Site Principal Investigator | Dr | Simon P | Hart | PhD | Hull, UK |
| Clinical Trials Research Unit (Statistics) | Ms | Hannah | Mather | MSc | Leeds, UK |
| Co-Investigators/grant holders | Professor | Małgorzata | Krajnik | PhD | Department of Palliative Care, Collegium Medicum in Bydgoszcz, Nicolaus Copernicus University in Toruń, Bydgoszcz, Poland |
| Site Principal Investigator | Dr | Silvia | Tanzi | PhD | Azienda USL-IRCCS di Reggio Emilia, Italy |
| Qualitative Researcher | Dr | Luca | Ghirotto | PhD | Azienda USL-IRCCS di Reggio Emilia, Italy |
| Other Clinicians | Professor | Charlotte E | Bolton | MD | Nottingham, UK |
| Site Principal Investigator | Dr | Piotr | Janowiak | PhD | Division of Pulmonology, Medical University of Gdańsk, Gdańsk, Poland |
| Site Project Manager | Dr | Elena | Turola | PhD | Azienda AUSL-IRCCS Reggio Emilia, Italy |
| Co-Investigators/grant holders | Professor | Julia M | Brown | MSc | Leeds, UK |
| Other BETTER-B Research Consortium Members |  |  |  |  |  |
| Site Principal Investigator | Dr | Emer | Kelly | Associate Clinical Professor | SVUH, Ireland |
| Other clinicians | Dr | Mirco | Lusuardi | MD | Azienda AUSL-IRCCS Reggio Emilia, Italy |
| Co-Investigators/grant holders | Dr | Rossella | Cianci | PhD | Universitia' Cattolica, Rome, Italy |
| Co-Investigators/grant holders | Professor | Ewa | Jassem | PhD | Division of Pulmonology, Medical University of Gdańsk, Gdańsk, Poland |
| Site Principal Investigator | Professor | Kathrin | Kahnert | PD Dr | University Hospital Muenchen, Germany |
| Patient and public involvement | Ms | Debs | Smith | BA | U.K |
| Health economics | Dr | Samantha | Smith | PhD | Trinity College Dublin |

## Acknowledgements

We are grateful to all the patients and caregivers who took part in this study. We also want to acknowledge the helpful advice and input from our patient and public involvement (PPI) group members and our partners from the European Lung Foundation, ELF, and European Respiratory Society.

We thank the BETTER-B independent critical friends and advisors (some also served on oversight committees): Professor Giovanni Apolone, the late Professor Randall Curtis, Professor Daisy J.A. Janssen, Professor Michael Kreuter. We thank the members of the independent data monitoring and safety committee (including Professor Magnus Ekstrom and Dr Pauline Kane), trial steering committee (Dr Brian Cassel, Dr Cinzia Brunelli, Dr Sharon Love, Professor Daisy Janssen, Professor Michael Kreuter, Professor Mogens Grønvold, the late Dr Sarah Booth,) and ethics advisory board (including Dr Wendy Prentice, the late Professor Randall Curtis).

We thank:

- Co-Sponsor Representative - UCD, Ireland: Dr Anna Malara
- Clinical Trials Research Unit - LEEDS, UK: Ms Emma Batman, Mr Jacob Burman, Mrs Claire Dimbleby, Mrs Fiona Walker, Ms Aaisha Ali
- Health Economics – TCD, Ireland: Ms Jingjing Jiang.
- Other clinicians, including recruiting clinicians and research nurses in particular: Mater Hospital, Ireland: Aoife Kelly, Dr Kate O’Reilly; Azienda USL-IRCCS di Reggio Emilia, Italy: Dr Alessandro Scarascia, Dr Martina Garofalo, Dr Sofia Taddei, Dr Francesco Menzella, Patrizia Ruggiero; UCSC, Italy: Dr Giovanni Gambassi; UMK, Poland: Dr Agnieszka Nowakowska-Arendt; University Hospital Munich, Germany: Sabine Streitwieser; Solingen, Germany: Anika Hain,; King’s College Hospital, UK: Paramjote Kaler; Nottingham University Hospital, UK: Emma Hadfield, Cathann Manderson; Nottingham, UK: Dr Vincent Crosby; Castle Hill Hospital, UK: Caroline Wright, Rachel Thompson, Rachel Flockton.
- Co-ordinators and researchers in particular: KCL, UK: Ms Chloe Nast; UCD, Ireland: KOELN, Germany: Dr Anne Pralong, Professor Raymond Voltz
- Patient and public involvement advisors, in particular: ERS, Switzerland: Ms Valerie Vaccaro.
